# Targeting NUCKS1 with a fragment of tRNA^Asn(GUU)^ of Chinese yew for the treatment of colorectal cancer

**DOI:** 10.1016/j.ncrna.2024.11.002

**Published:** 2024-11-12

**Authors:** Kai-Yue Cao, Da Zhang, Long-Bo Bai, Tong-Meng Yan, Yan Chen, Yu-Yang Jiang, Zhi-Hong Jiang

**Affiliations:** a State Key Laboratory of Quality Research in Chinese Medicine, Macau University of Science and Technology, Avenida Wai Long, Taipa, Macau SAR, China; b School of Pharmacy, Shenzhen University Medical School, Shenzhen University, Shenzhen, Guangdong, China; c State Key Laboratory of Chemical Oncogenomics, Tsinghua Shenzhen International Graduate School, Shenzhen, Guangdong, China

**Keywords:** NUCKS1, CRC, tRFs, PI3K-Akt, Chinese yew, Taxus

## Abstract

Despite the discovery of numerous oncogenes in colorectal cancer (CRC), the development of associated drugs is limited, posing a significant challenge for CRC treatment. Identification of novel druggable targets is therefore crucial for the therapeutic development of CRC. Here, we report the first investigation on therapeutics targeting the potent oncogene NUCKS1 to suppress cancer progression. NUCKS1-orientated bioinformatics screening of NUCKS1 inhibitors from our library of tRNA fragments originated from medicinal plants identified tRF-T36, a 5′ tRNA fragment of tRNA^Asn(GUU)^ of Chinese yew (*Taxus chinensis*), exhibiting stronger inhibitory effects than taxol against CRC progression. Mechanistically, tRF-T36 binds directly to the 3′ UTR of NUCKS1 mRNA to downregulate its expressions *via* RNAi pathway. High-throughput RNA sequencing indicated that the downregulated NUCKS1 induced by tRF-T36 further inhibits PI3K/Akt pathway, as verified by the significantly efficacy decrease of tRF-T36 mimic in co-treatment with 740Y-P, an agonist of PI3K/Akt pathway. Collectively, our findings emphasize the importance of NUCKS1 as a promising druggable target for CRC. Furthermore, the present study provides the first siRNA sequence, tRF-T36 mimic, as small RNA drug candidate, thereby shedding light on CRC therapeutics.

## 1 Introduction

As a significant global public challenge in health, colorectal cancer (CRC) is the third most frequently diagnosed cancer and ranks second in cancer-related mortality worldwide [1]. Current therapeutic strategies for CRC include irinotecan, oxaliplatin, fluoropyrimidines (5-fluorouracil and capecitabine), EGFR antibodies (cetuximab and panitumumab), and VEGF antibodies (bevacizumab and ziv-aflibercept) [2]. However, unexpected limitations of the above therapeutics frequently occur in terms of their target genes and clinical chemotherapeutic applications. For instance, although EGFR is frequently overexpressed to promote tumor progression [3], resistance to its treatment continues to be a major obstacle for CRC patients [4]. Mutations in MAPK such as KRAS or BRAF were reported to frequently impact the therapeutic outcomes of MAPK inhibitors [5,6]. In addition, the dysregulated Wnt signaling pathway, driven by mutations in APC, CTNNB1, or AXIN2, promotes uncontrolled cell proliferation in CRC [[7], [8], [9]]. Therefore, more novel druggable targets should be explored to benefit the development of innovative therapeutic strategies to improve CRC therapy.

Nuclear casein kinase and cyclin-dependent kinases substrate 1 (NUCKS1) has been revealed with a high expression level in various solid tumors [10]. It occupies a significant position in the complex landscape of tumor development and progression, displaying distinct roles in different cancer types and illustrating its multifaceted impact. In breast cancer, for example, NUCKS1 acts as a powerful promoter of tumor growth and invasiveness, facilitating cellular proliferation and invasion [11,12]. Similarly, in hepatocellular carcinoma, NUCKS1 plays a crucial role in conferring resistance to chemotherapy, complicating treatment strategies and limiting therapeutic options [13]. In lung cancer, NUCKS1 drives the relentless invasion and proliferation of cancer cells, exacerbating the disease aggressiveness [14]. In gastric cancer, NUCKS1 significantly contributes to tumor progression through mTOR-Beclin1 pathway, leading to poor patients’ outcome [15]. The intricate mechanisms through which NUCKS1 exerts its influence involve extensive gene expression alterations and the activation of downstream signaling cascades, collectively fostering conditions favorable for uncontrolled tumor growth and metastasis. Additionally, NUCKS1 in CRC tumors is overexpressed, indicating its potential role as a prognostic marker and therapeutic target of CRC [16,17]. These evidences suggest that targeting NUCKS1 holds promise for cancer treatment. However, to our best knowledge, targeting NUCKS1 for cancer treatment has not been investigated.

Fragments derived from transfer RNA, such as tRNA-derived fragments (tRFs) and tRNA halves, are naturally degraded RNA sequences by specific nucleases cleaved from the 5′ and 3′ ends of tRNAs that are stably expressed in all living organisms [18]. Endogenous tRF identified from breast cancer plays a regulatory role through the RNAi pathway to target YBX1, resulting in suppressing breast cancer [19]. Another endogenous 5′-tRNA half was revealed to inhibit the breast cancer progression by targeting FZD3 [20]. In our previous studies, a tRF library derived from medicinal plants have been built for screening pharmacologically-active RNA sequence. For instance, a 5′-tRF derived from Chinese yew has been identified to suppress ovarian cancer by targeting TRPA1 [21]. Additionally, a 5′ tRF derived from ginseng has been discovered to protect the heart from ischemia/reperfusion injury [22], and a Ganoderma-derived 3′ tRNA half has shown broad inhibitory effects on a variety of cancer cell types [23]. Therefore, it was estimated that exogenous tRF might be a promising small RNA silencing oncogene such as NUCKS1.

In this study, a bioinformatics-based screening of NUCKS1 inhibitors identified tRF-T36 (a tRNA-derived fragment of Chinese yew, *Taxus chinensis*) from our tRF library as a putative inhibitor of NUCKS1. *In vitro* and *in vivo* experiments confirmed its strong inhibitory effects on CRC progression *via* RNAi pathway. High-throughput RNA sequencing revealed that tRF-T36 targets NUCKS1 to inhibit PI3K/Akt pathway, which was further validated through a rescue experiment using 740Y-P, a PI3K/Akt agonist, indicating the potential of tRF-T36 as a siRNA drug candidate for CRC therapy.

## 2 Materials and methods

### 2.1 Chemicals and reagents

Taxol (purity>98 %) was obtained from AdooQ Bioscience (China). F-12K medium and MTT [3-(4,5-dimethylthiazol-2-yl)-2,5-diphenyltetrazolium bromide] were obtained from Thermo (U.S.A.). 740Y-P was sourced from MedChemExpress (U.S.A.). Penicillin, RPMI 1640 medium, streptomycin, and fetal bovine serum (FBS) were acquired from Gibco (New Zealand). Deionized water was produced by Millipore Milli-Q Plus system (Millipore, U.S.A.). All RNAs used in this work were purchased from Biosyntech Co., Ltd. (China).

### 2.2 TCGA data analysis

RNA sequencing data of clinical samples for various cancers were retrieved from TCGA database (https://portal.gdc.com). The latest release of GTEx database was utilized through GTEx data portal website (https://www.gtexportal.org/home/datasets). R software v4.0.3 (R Foundation for Statistical Computing, Austria) was employed for statistical analysis. P value < 0.05 is considered as statistical significance.

### 2.3 Cell culture

HEK293T human embryonic kidney cell line, HCoEpiC human colon epithelial cell line, human CRC cell lines LoVo, HCT-8, and its taxol-resistant strain HCT-8/T were obtained from American Type Culture Collection (ATCC). All cells were maintained in RPMI 1640 medium except for LoVo cells cultured in F-12K medium (Gibco). The above cells were maintained at 37 °C in a humidified atmosphere with 5 % CO_2_, while medium was supplemented with 10 % FBS and 1 % penicillin and streptomycin.

### 2.4 Liposomal transfection and cytotoxic assay

Transfection was carried out by employing Lipofectamine RNAiMAX Transfection Reagents (Thermo, U.S.A.). Specifically, cells were seeded with a density of 5 × 10^3^ cells/well in 96-well plates. After 20 h, medium was changed to RNA samples, taxol or blank medium. After treatment for 48 h, MTT solution was added to a final concentration at 1 mg/mL, followed by incubated for 4 h at 37 °C. Finally, all solvents were removed and DMSO were added to measure the absorbance at 570 nm using a SpectraMax microplate reader (Molecular Devices, U.S.A.). IC_50_ values were determined using GraphPad Prism software (U.S.A.). The experiment was conducted in triplicate. The results are expressed as means ± standard deviation.

### 2.5 Colony formation

The colony formation was conducted with minor modifications [24]. In brief, cells were seeded with a density of 500 cells/well in 24-well plates filled with culture medium. The medium was replaced with RNA samples at various concentrations after 20 h, along with liposomes or taxol. After treatment for 48 h, mediums were changed to fresh culture medium. After culture for another 14 days, paraformaldehyde solution (Beyotime, China) were used to fix the cells, followed by stained with crystal violet (Beyotime, China) and the images were captured.

### 2.6 Wound healing assay

5 × 10^5^ CRC cells were seeded into 24-well plates to confluence over 20 h. A scratch was made using a 200 μL sterile pipette tip, followed by replaced the solvents to medium containing RNA samples, liposomal transfection agents or taxol. Under microscope (Leica Microsystems, Germany) at 10 × magnification, cells were observed and the images were captured at 0 and 24 h. The wound area was quantified and ImageJ software was used to calculate the wound healing rate.

### 2.7 Caspase-3 activity assay

A caspase-3 colorimetric assay kit (Sigma, U.S.A.) was employed to determine the caspase-3 activity. HCT-8 and LoVo cells with the density of 2 × 10^6^ were seeded onto dishes. After 20 h, the medium was changed to solvents contained either tRF-T36 mimic or taxol. The cells were collected after 18 h by centrifugation at 800×*g* for 5 min. Cell lysates were then combined with Ac-DEVD-pNA which is colorimetric peptide substrates and incubated for 20 h, followed by measured the absorbance at 405 nm.

### 2.8 High-throughput RNA sequencing

Cellular total RNA was extracted using Trizol method (Thermo). OligodT was employed to enrich mRNAs following the instructions of the NEBNext Poly(A) mRNA Magnetic Isolation Module (NEB, U.S.A.). The mRNAs were then fragmented to approximately 200 bp. Subsequently, cDNA synthesis, adaptor ligation, and low-cycle enrichment were carried out using NEBNext Ultra RNA Library Prep Kit. Agilent 2200 TapeStation and Qubit (Thermo) were employed to evaluate the purified library products. Paired-end sequencing was performed using an Illumina sequencer (Illumina, U.S.A.) with 150 bp reads.

### 2.9 Differentially expressed genes (DEG) analysis

To visualize the expression patterns of DEGs in different groups, a hierarchical clustering analysis was carried out using the R language package gplots based on TPM values. The clusters were assigned different colors to represent their clustering information, such as genes with similar expression patterns within the same group, indicating potential functional similarities or involvement in the same biological processes.

### 2.10 Quantitative real-time PCR

Reverse transcription of extracted RNA was carried out using GoScript Reverse Transcription System (Promega, U.S.A.). Quantitative real-time PCR was conducted on a ViiATM 7 system (Life Technologies, U.S.A.) utilizing the GoTaq qPCR Master Mix (Promega, U.S.A.). PCR primers were commercially sourced from BGI Genomics Co., Ltd. (China, Table S1). The data are expressed as the average fold change values normalized to GAPDH using the 2^−ΔΔCT^ method. Each experiment was conducted in triplicate, and the results are presented as means ± standard deviation.

### 2.11 Western blotting assay

RIPA reagent (Cell Signaling Technology, U.S.A.) supplemented with inhibitor cocktails involving protease and phosphatase (Roche, Switzerland) were used to extract proteins, followed by determined the protein concentrations using BCA protein assay kit (Thermo). 10 % SDS-PAGE was applied for protein separation, followed by transferred onto nitrocellulose membranes and blocked by 5 % bovine serum albumin (Thermo). Subsequently, the membranes were added with primary antibodies against NUCKS1 (#PA5-26535, 1:1000 dilution, Thermo) or GAPDH (#5174, 1:1200 dilution, Cell Signaling Technology) overnight for incubation. Following TBST washing, the membranes were incubated with a fluorescent secondary antibody (#ab216773, 1:8000 dilution, Abcam). Band intensities were quantified using ImageJ software and normalized to GAPDH. A scramble double-stranded RNA of tRF-T36 mimic (Forward: 5′-GCUGGGCUAUCAACGUCUCACA-3′, Reverse: 5′-CGACCCGAUAGUUGCAGAGUGU-3′) was employed as a negative sequence. The experiment was conducted in triplicate, and the results are presented as means ± standard deviation.

### 2.12 Dual-luciferase reporter assay

The pmiR-RB-REPORT vector (Ribobio, China) was utilized to introduce the wild-type or mutant tRF-T36 binding sites into the Xhol and Notl restriction sites within the 3′ UTR of NUCKS1 mRNA. HEK293T cells were co-transfected with luciferase reporter vectors and either the tRF-T36 mimic or a non-target control. After treatment for 48 h, firefly and renilla luciferase activities were measured using the Dual-Glo Luciferase Assay System (Promega) and the GLOMAX 96 spectrophotometer (Promega). The scramble dsRNA of tRF-T36 mimic used in the western blotting assay was served as a negative control. Each experiment was conducted in triplicate.

### 2.13 Nanoparticles preparation

Appropriate HKP (provided by Dr. Lu, Sirnaomics, U.S.A.) powders were weighed and dissolved in RNase-free water to achieve a concentration of 240 μg/mL and incubated for 20 h at 4 °C, followed by mixed with tRF-T36 mimic solutions (80 μg/mL) with equal volume for 30 min at room temperature.

### 2.14 *In vivo* anti-tumor experiment

Animal experiment was carried out under the guidelines established by the Institutional Animal Care and Use Committee of Macau University of Science and Technology, and it received approval from the Ethics Committee of the same institution (protocol code MUSTARE-003-2020). All mice were maintained under pathogen-free environment in the animal facility at Macau University of Science and Technology.

Subcutaneous injections of HCT-8 cells with density of 4.0 × 10^6^ were performed under the armpits of 6- to 8-week-old female Balb/c nude mice around weight of 18–20 g (Zhuhai Bestest Bio-Tech Co., Ltd, China). When tumors grew to 50 mm^3^, intratumoral injections of tRF-T36 mimic at 2.5 mg/kg encapsulated with HKP nanoparticles were carried out every three days. Taxol (5 mg/kg) was selected as positive control, while HKP served as a blank control. On day 22, all mice were sacrificed, and digital caliper was used to measure tumor diameters at their maximum length and width. The volume of tumors was then calculated using the formula: Volume = width^2^ × length/2. Statistical analysis was performed using GraphPad.

### 2.15 Histological analysis

Mouse tissues were fixed in PFA solutions (4 %) and subsequently implanted by paraffin. For immunohistochemistry (IHC), the slides were dewaxed using xylene and rehydrated through a graded series of ethanol concentrations. Antigen retrieval was performed by sodium citrate (10 mM, pH = 6.0) incubation at 95 °C for 30 min. The slides were then treated with 3 % H_2_O_2_, followed by PBS washing for three times and blocking with 5 % BSA. Subsequently, the slides were incubated with primary antibody against ki67 (#ab279653, 1:1000 dilution, Abcam) or PCNA (#ab92552, 1:1000 dilution, Abcam), followed by incubation with a GTVison secondary antibody (#GK600710A, 1:1000, Gene Tech, China). Antibody complexes were visualized using 3,3′-diaminobenzidine and subsequently stained with hematoxylin. Additionally, paraffin sections were cut into 3–5 μm, followed by stained with hematoxylin and eosin.

### 2.16 Statistical analysis

The data are presented as means ± SD. Statistical analysis was carried out using GraphPad software, utilizing multiple two-tailed student's t-tests or two-way ANOVA with subsequent post hoc analysis. Significance levels in all figures are defined as follows: P < 0.05 (∗), P < 0.01 (∗∗), P < 0.001 (∗∗∗), and P < 0.0001 (∗∗∗∗).

## 3 Results

### 3.1 NUCKS1 is overexpressed in multiple cancer patients

**Fig. 1:**
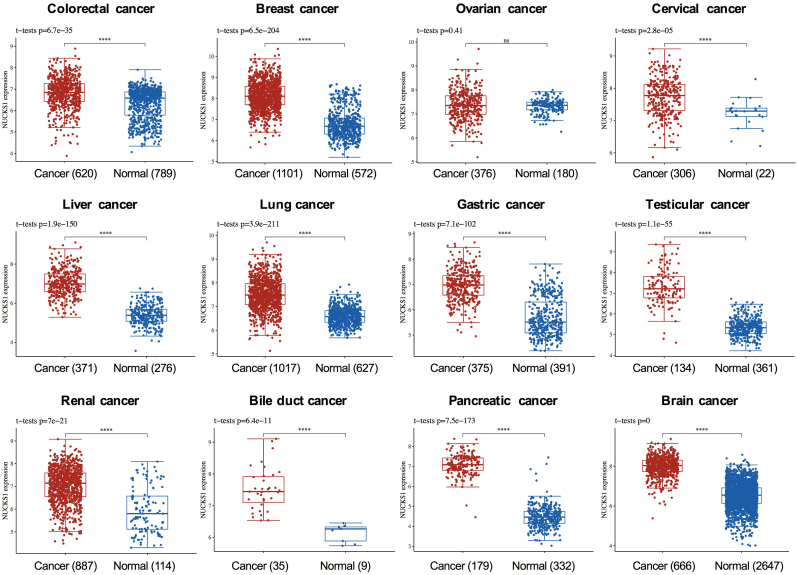
**Expression levels of NUCKS1 in 12 kinds of cancer using TCGA data**. ∗∗∗∗*P* < 0.0001.

To determine the overexpression of NUCKS1 in tumors, genomic data was statistical analyzed and displayed in Fig. 1, revealing a significant upregulation of NUCKS1 levels in 12 selected tumors when compared to their cognate adjacent tumor tissues or normal tissues, with the exception of ovarian cancer. This observation aligns with the current understanding of NUCKS1 as an oncogene in CRC, and suggests its broader involvement in related cancer types [10]. Consequently, these findings speculated NUCKS1 as a potential target for CRC treatment.

### 3.2 tRF-T36 binds to 3′ UTR of NUCKS1 mRNA and inhibits its expressions through RNAi pathway

**Fig. 2:**
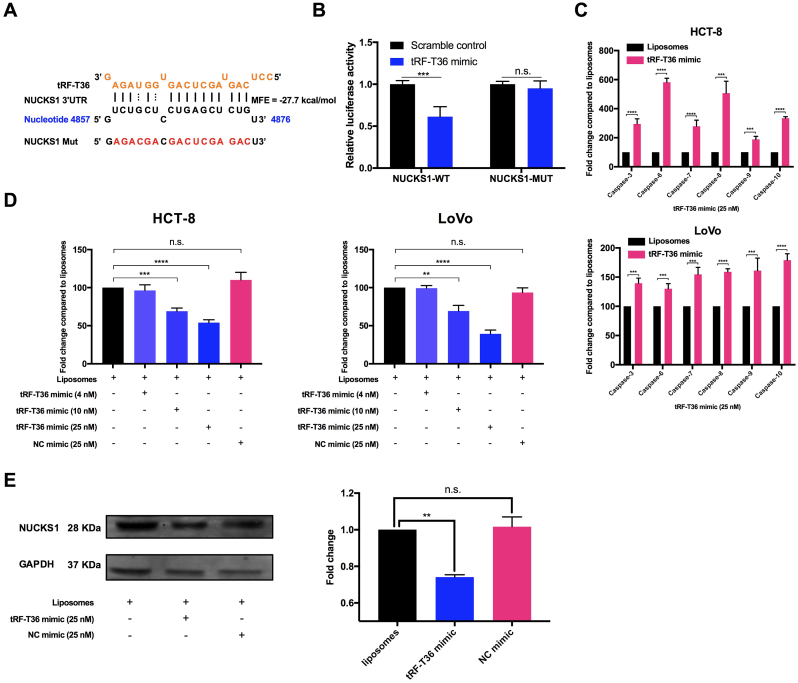
**tRF-T36 targets NUCKS1 and inhibits its expressions on both mRNA and protein levels through RNAi pathway.** (**A**) Putative binding sites of tRF-T36 on the 3′ UTR region of NUCKS1 mRNA. (**B**) The relative luciferase activities were detected by transfecting pmiR-RB-Report h-NUCKS1-WT or pmiR-RB-Report h-NUCKS1-MUT and tRF-T36 mimic or its scramble mimic into HEK-293T cells. (**C**) Quantitative real-time PCR analysis of caspase-family genes associated with apoptosis in HCT-8 and LoVo cells treated with tRF-T36 mimic. (**D**) tRF-T36 mimic suppressed the mRNA levels of NUCKS1 in a dose-dependent manner; (**E**) Western blotting analysis of protein levels of NUCKS1 in HCT-8 cells treated by tRF-T36 mimic at 25 nM. Data are shown as the means ± SDs of three independent experiments. ∗∗*P* < 0.01, ∗∗∗*P* < 0.001, ∗∗∗∗*P* < 0.0001.

Based on the tRF library built in our previous work [[21], [22], [23]], in-depth bioinformatics analysis was carried out to screen tRF targeting NUCKS1 [25]. As a result, tRF-T36, which is derived from 5′ termini of tRNA^ASN(GUU)^ of Chinese yew in 22-nucleotide length, specifically bind to the 3′ UTR mRNA of NUCKS1 in a low minimum free energy (MFE) of −27.7 kcal/mol with the complementary rate over 72 % (Fig. 2A). Moreover, the results in dual-luciferase reporter assay showed that the tRF-T36 mimic markedly decreased the luciferase activity of the NUCKS1-WT reporter vector. Conversely, the NUCKS1-MUT reporter gene vector showed no significant change in luciferase activity (Fig. 2B). These findings verified that the interaction between tRF-T36 and NUCKS1 mRNA happens *via* the predicted binding sites.

siRNA functions through RNA interference pathway would silence or downregulate specific genes in cancer cells, subsequently triggering classical cellular apoptosis and inducing cell death [26]. Numerous evidences have been raised that the apoptosis-related gene family Caspases can serve as significant biomarkers [27]. Thus, to confirm the impact of tRF-T36 mimic in occurrence of apoptosis in CRC cells, the expressions of caspase-3, -6, -7, -8, -9 and -10 were analyzed by qPCR analysis. The results revealed significant upregulation of all the above genes in both CRC cells treated with tRF-T36 mimic at a concentration of 25 nM, with a more induction fold observed in HCT-8 cells (Fig. 2C).

Furthermore, qPCR analysis was employed to verify the predicted results. It was observed that tRF-T36 mimic dose-dependently downregulated NUCKS1 mRNA expressions in both HCT-8 and LoVo cells (Fig. 2D). Using a double-stranded RNA in our previous work as a negative control (NC) mimic [21], no significant change of NUCKS1 mRNA was observed in NC mimic-treated CRC cells. Furthermore, western blotting assay addressed that NUCKS1 significantly decreased in tRF-T36 mimic-transfected HCT-8 cells, while NC sequence did not exhibit such effect (Fig. 2E). These results indicate that tRF-T36 binds to the predicted sites within the 3′ UTR mRNA of NUCKS1, thus downregulating its expression.

### 3.3 tRF-T36 mimic suppresses the proliferation, colony formation and migration of CRC cells *in vitro*

**Fig. 3:**
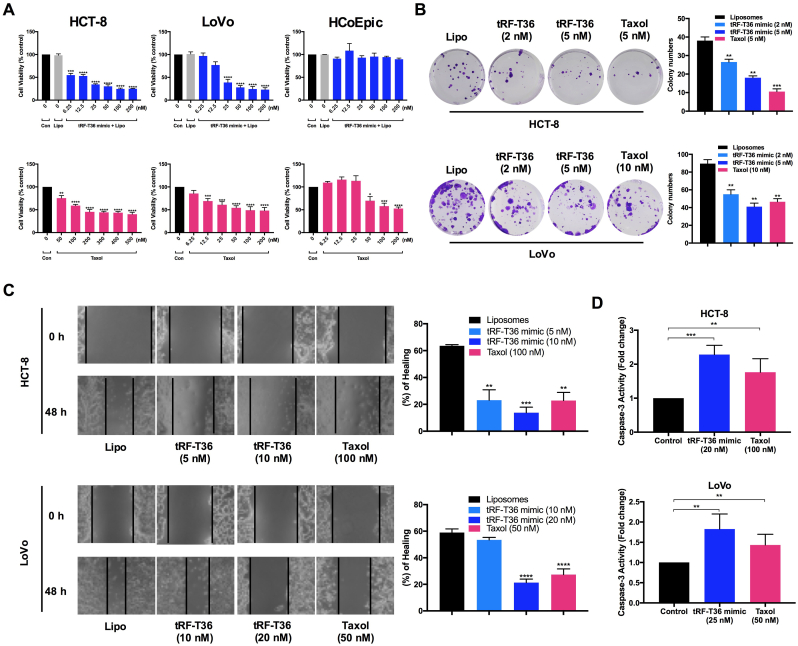
**tRF-T36 mimic inhibits human CRC cell proliferation, colony formation and migration.** (**A**) Comparison of the dose-dependent effects of tRF-T36 mimic and taxol against HCT-8, LoVo, and HCoEpiC cells. (**B**) Clonogenic assay of tRF-T11 mimic on HCT-8 and LoVo cells. (**C**) Wound healing assay of tRF-T36 mimic on HCT-8 and LoVo cells. (**D**) Determination of caspase-3 activity in HCT-8 and LoVo cells treated with tRF-T36 mimic. Data are presented as the means ± SDs of three independent experiments. ∗∗*P* < 0.01, ∗∗∗*P* < 0.001, ∗∗∗∗*P* < 0.0001.

As depicted in Fig. 3A, the tRF-T36 mimic exhibits significant cytotoxicity with low IC_50_ values of 10.0 nM in HCT-8 cells and 27.54 nM in LoVo cells, respectively. In comparison, taxol as a positive control has much higher IC_50_ values (216.5 nM in HCT-8 and 103.8 nM in LoVo), which is 20-fold and 3.7-fold higher than those of tRF-T36 mimic, indicating the remarkable cytotoxicity of tRF-T36 mimic against CRC cells. Meanwhile, cytotoxic effects of tRF-T36 mimic were tested on normal human colon epithelial cell line HCoEpiC, revealing no significant cytotoxicity under the concentration of 200 nM, while taxol exhibited strong cytotoxicity with an IC_50_ value of 167.0 nM. It is worth noting that tRF-T36 mimic exhibited similar cytotoxicity on taxol-resistant HCT-8 cells (IC_50_ value = 9.49 nM), which is 163-time less than that of taxol (1.55 μM) (Fig. S1).

Furthermore, the efficacy of tRF-T36 mimic in inhibiting colony formation was determined on both two CRC cells. The results demonstrated a dose-dependent suppression of clonogenic ability in both cell lines, similar to the positive control taxol (Fig. 3B). In addition, since migration is a critical factor in CRC treatment, a wound healing experiment was carried out. As shown in Fig. 3C, at 48 h, HCT-8 cells treated with tRF-T36 mimic displayed a significantly lower wound healing rate (WHR) of 23.1 ± 7.6 % at 5 nM and 13.7 ± 4.1 % at 10 nM compared to liposomal control (63.5 ± 0.9 %), while taxol-treated cells exhibited a comparable one (22.8 ± 6.1 %). In LoVo cells, tRF-T36 mimic-treated cells exhibited a significantly lower WHR of 53.5 ± 1.8 % at 10 nM and 21.3 ± 2.6 % at 20 nM compared to liposomal control (58.9 ± 2.7 %), while taxol-treated cells exhibited a comparable one (27.3 ± 4.2 %). Furthermore, the caspase-3 activity in CRC cells was determined by ELISA method since it is one of the key biomarkers of apoptosis [28]. The results demonstrated that tRF-T36 mimic significantly increased caspase-3 activity by approximately 2-fold after 18 h treatment in both CRC cell lines (Fig. 3D), which correlates with the results from qPCR assay. Overall, the above findings indicated that tRF-T36 mimic strongly suppresses the proliferation, colony formation, and migration in CRC cells.

### 3.4 tRF-T36 mimic might trigger PI3K/Akt pathway through targeting NUCKS1

**Fig. 4:**
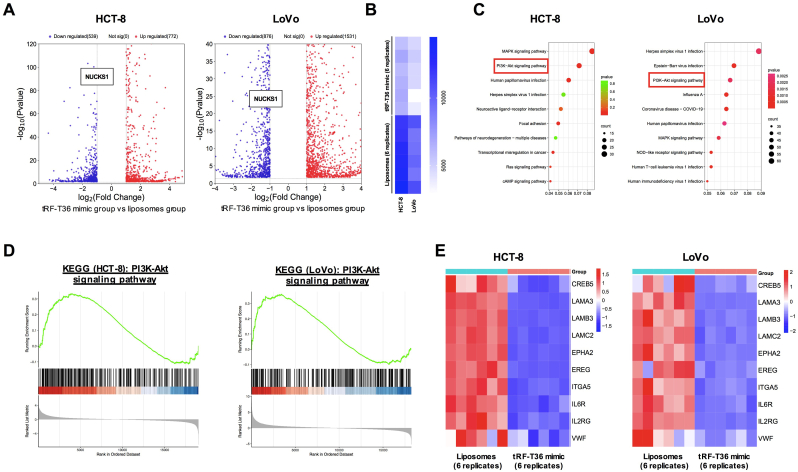
**High-throughput RNA sequencing.** (**A**) Volcano plot of differentially expressed genes in HCT-8 and LoVo cells treated with tRF-T36 mimic or empty liposomes. (**B**) Heatmap of NUCKS1 gene expressions in CRC cells treated with tRF-T36 mimic or empty liposomes, n = 6 for each group. (**C**) Dot plot of the KEGG pathway enrichment analysis of HCT-8 and LoVo cells treated with tRF-T36 mimic. The horizontal axis represents the gene ratio, while the vertical axis represents the enriched pathway name. The color scale indicates different thresholds of the p-value, and the size of the dot indicates the number of genes corresponding to each pathway. (**D**) GSEA analysis showed that the enriched PI3K-Akt was suppressed in tRF-T36 mimic-treated CRC cells. (**E**) Heatmap of key genes expressions in PI3K-Akt pathway between CRC cells treated with tRF-T36 mimic (right) or empty liposomes (left).

Since NUCKS1 was suggested to function as an oncogene in CRC, its potential signaling pathway is critical to better understand the in-depth mechanisms of tRF-T36 mimic. Thus, a high-throughput RNA sequencing using tRF-T36 mimic transfected CRC cells (n = 6) and liposomes-treated CRC cells (n = 6) was performed to identify differentially expressed genes (DEG). Hierarchical bi-clustering analysis demonstrated the significant gene signatures in both two CRC cells between tRF-T36 mimic transfected groups and liposome-treated groups (Fig. S2). DEG analysis showed that in HCT-8 cells, 539 significant genes were downregulated in tRF-T36 mimic transfected group (log2FC > 1, P < 0.05), while 772 genes showed relatively higher expression (log2FC < −1, P < 0.05). In LoVo cells, 876 genes were significantly downregulated in tRF-T36 mimic transfected group (log2FC > 1, P < 0.05), while 1531 genes were relatively upregulated (log2FC < −1, P < 005, Fig. 4A). Notably, the mRNA expression of NUCKS1 in CRC cells treated with tRF-T36 mimic was substantially downregulated, which is in agreement with the qPCR results (Fig. 4B). Furthermore, Kyoto Encyclopedia of Genes and Genomes (KEGG) analysis was employed to analyze the pathways correlated with NUCKS1. The results indicated that tRF-T36 mimic treatment in CRC cells had significant impact on several molecular pathways. Among which, PI3K/Akt possessed as the most significantly enriched pathway in both HCT-8 and LoVo cells, which is regulated by NUCKS1 and involved in tumor progression (Fig. 4C). Consistently, gene set enrichment analysis (GSEA) demonstrated a notable positive correlation between NUCKS1 and the PI3K-Akt pathway (Fig. 4D). Moreover, analysis of the RNA-seq data revealed significant downregulation of major molecules involved in the PI3K-Akt pathway, including CREB5, LAMA3, LAMB3, LAMC2, EPHA2, EREG, ITGA5, IL6R, IL2RG, and VWF, in tRF-T36 mimic-treated cells (Fig. 4E). Therefore, PI3K/Akt pathway might be considered as a candidate downstream signaling pathway of NUCKS1 inhibited by tRF-T36 mimic.

### 3.5 PI3K/Akt pathway mediates the inhibition effects of NUCKS1 by tRF-T36 mimic on CRC cells

**Fig. 5:**
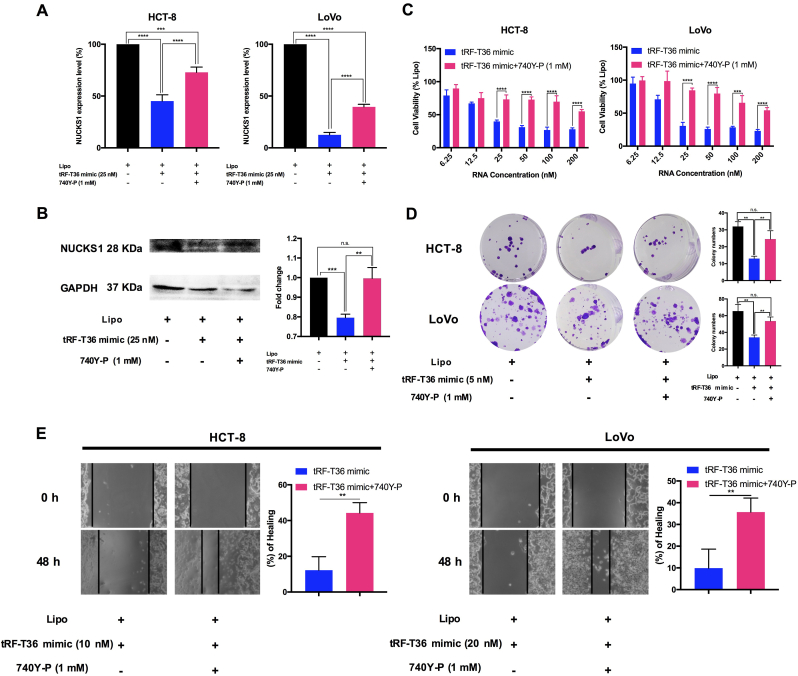
**Rescue experiment by co-treatment of tRF-T36 mimic and 740Y-P on CRC cells.** NUCKS1 mRNA levels (**A**), protein levels (**B**), IC_50_ values (**C**), clonogenic ability (**D**) and wound healing ability (**E**) in CRC cells co-treated with tRF-T36 mimic and 740Y-P were upregulated compared to tRF-T36 mimic treatment alone. Data are shown as the means ± SDs of three independent experiments. ∗∗*P* < 0.01, ∗∗∗*P* < 0.001, ∗∗∗∗*P* < 0.0001.

To verify whether the inhibition effects of tRF-T36 mimic on CRC cells were mediated by PI3K/Akt, a rescue experiment was carried out with the employment of 740Y-P which is an agonist of PI3K-Akt pathway [29]. Compared to the treatment of tRF-T36 mimic alone, the results demonstrated that co-treatment of tRF-T36 mimic and 740Y-P significantly increased NUCKS1 mRNA levels in CRC cells (Fig. 5A). Also, NUCKS1 protein level in HCT-8 cells significantly upregulated in the presence of co-treatment compared to tRF-T36 mimic alone (Fig. 5B). Furthermore, the combination of tRF-T36 mimic and 740Y-P exhibited a similar trend of inhibitory effects on CRC cells compared to tRF-T36 mimic alone (Fig. 5C), as demonstrated by the substantial rise in the IC_50_ value (24.55 nM–460.2 nM in HCT-8 cells, 24.14 nM–221.2 nM in LoVo cells). In clonogenic assay, it was observed that the clonogenic ability of CRC cells with co-treatment were significantly suppressed compared to tRF-T36 mimic alone (Fig. 5D). Furthermore, both the two CRC cells with co-treatment of tRF-T36 mimic and 740Y-P exhibited a significantly higher wound healing rate (44.3 % ± 5.72 % for HCT-8 cells and 35.6 % ± 6.4 % for LoVo cells) compared to tRF-T36 mimic alone (12.2 % ± 7.58 % for HCT-8 cells and 9.9 % ± 8.7 % for LoVo cells) (Fig. 5E). In summary, the above findings demonstrate that the suppressive-effect of tRF-T36 mimic on CRC cells are modulated by PI3K/Akt pathway, which is in agreement with RNA-seq results (Fig. 4).

### 3.6 tRF-T36 mimic significantly suppresses CRC progression and targets NUCKS1 *in vivo*

**Fig. 6:**
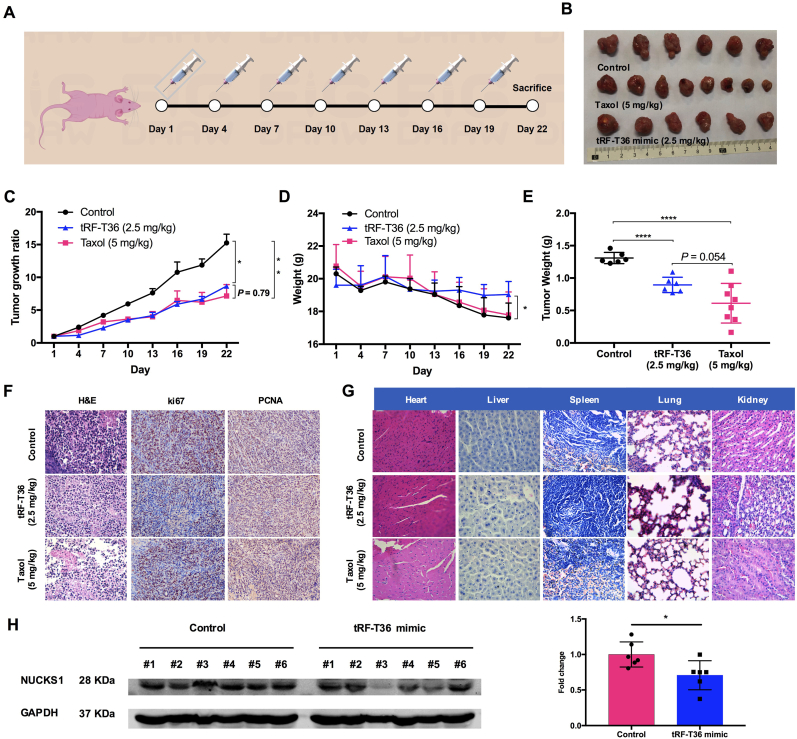
**tRF-T36 mimic suppresses CRC tumor growth and downregulates NUCKS1 expression *in vivo*.** (**A**) Workflow of animal experiment. (**B**) Each tumor in all groups (n = 6 for HKP control and tRF-T36 mimic treatment group, n = 8 for taxol treatment group). (**C**) Growth rate of tumors treated with HKP encapsulated tRF-T36 mimic, taxol, or HKP alone as control. (**D**) Plot of body weight change among all groups. (**E**) Weight of tumor removed from the mice at day 22. (**F**) Representative images of hematoxylin and eosin staining, ki67, and PCNA immunohistochemistry of tumor samples at 200 × magnification. (**G**) Representative images of hematoxylin and eosin stained major organs in HCT-8 xenografts nude mice. (**H**) Expression of NUCKS1 in each tumor tissue from control group and tRF-T36 mimic-treated group. Representative Western blot images are presented in the left panel. Data are shown as the means ± SDs of three independent experiments. ∗∗*P* < 0.01, ∗∗∗*P* < 0.001, ∗∗∗∗*P* < 0.0001.

The tRF-T36 mimic was encapsulated using a histidine-lysine polymer (HKP) to facilitate its efficient delivery *in vivo*. To evaluate the potential of the tRF-T36 mimic in inhibiting colorectal cancer growth, an HCT-8 cell xenograft tumor model was employed. Tumors reaching 50 mm^3^ were intratumorally injected with encapsulated tRF-T36 mimic (2.5 mg/kg), taxol (5.0 mg/kg), or blank HKP once every three days (Fig. 6A). The groups receiving treatment demonstrated a significantly lower tumor progression rate (Fig. 6B and C). Notably, tRF-T36 mimic did not significantly impact animals’ body weight. In contrast, both the group treated with taxol and HKP exhibited a significant reduction in body weight (Fig. 6D). Both the tRF-T36 mimic-treated group and the taxol-treated group demonstrated a similar degree of reduction in tumor size, with approximately 50 % decrease in volume and weight (Fig. 6C–E). Notably, dosage of tRF-T36 mimic used was only half of that of taxol, yet it achieved no significant difference tumor-suppressive effects *in vivo*. Furthermore, the IHC results demonstrated that Ki67 and PCNA levels were significantly reduced in tumors subjected to tRF-T36 mimic or taxol treatment, indicating that the tRF-T36 mimic effectively inhibits CRC progression (Fig. 6F). Moreover, both morphological images (Fig. S3) and pathological analysis (Fig. 6G) indicated no significant histological variations in major tissues was observed when comparing tRF-T36 mimic-treated group to the control group, indicating the safety of tRF-T36 mimic treatment.

Furthermore, NUCKS1 expressions in tumors were measured by western blotting assay. The findings demonstrated that NUCKS1 protein levels in the tumors were significantly reduced in tRF-T36 mimic-treated mice other than those in the control group (Fig. 6H), which is consistent with the *in vitro* findings. Thus, it can be concluded that tRF-T36 mimic suppresses human colorectal cancer growth *via* targeting NUCKS1 both *in vitro* and *in vivo*.

## 4 Discussion

The tendency of CRC to metastasize and recur presents significant challenges, limiting the effectiveness of current chemotherapy treatments in clinical settings [30]. Clinically, CRC exhibits the ability to metastasize to distal organs, resulting in poor prognoses [31]. Meanwhile, CRC often relapses even after successful initial treatments, underscoring the need for therapies capable of effectively targeting and preventing metastasis and recurrence [32]. Unfortunately, although numerous targets have been discovered in CRC, the challenges mentioned above still exist. Through bioinformatics-based screening of NUCKS1 inhibitors from our library of tRF originated from medicinal plants, in this study, the potential therapeutic effects of a tRF-T36 mimic were investigated. The tRF-T36 mimic not only inhibited CRC cell proliferation, but also exhibited a significant effect on the wound healing ability of CRC cells, suggesting its potential efficacy. Notably, the inhibitory effect of the tRF-T36 mimic was observed equally in both HCT-8 cells and its taxol-resistant strain, demonstrating its potential as a therapeutic option on drug-resistant tumors, which is a commonly challenge for small molecular drugs such as taxol.

The PI3K-Akt pathway has been demonstrated as a critical player in cancer progression, including CRC [33,34]. This pathway is crucial in modulating various cellular processes, involving cell proliferation, survival, angiogenesis, metabolism, etc. [35]. Dysregulation of PI3K-Akt pathway is in agreement with the pathogenesis of multiple cancers, making it an attractive target for therapeutic interventions. PI3K-Akt pathway would be activated through multiple mechanisms such as genetic alterations in PI3K or Akt genes and the abnormal activation of upstream receptor tyrosine kinases [36]. Moreover, crosstalk between the PI3K-Akt pathway and other key signaling pathways like RAS-MAPK and Wnt-*β*-catenin, further contributes to the complex interplay involved in cancer progression [37]. Unfortunately, despite significant research efforts, the translation of PI3K-Akt pathway inhibitors into clinical treatments has encountered challenges such as mutations [38]. In this study, the tRF-T36 mimic effectively targeted and downregulated NUCKS1, a molecule in the PI3K-Akt pathway [39], resulting in successful suppression of CRC progression even at low dosages. Furthermore, considering the overexpression of NUCKS1 in multiple types of tumor, the tRF-T36 mimic could potentially have a broad-spectrum application in cancer therapy. Further investigations on the downstream signaling pathway of NUCKS1 inhibited by tRF-T36 mimic would be carried out in our future work.

Application of small-interfering RNA (siRNA) as a therapeutic tool for gene silencing has shown great promise in multiple fields such as oncology [40]. However, a crucial consideration when utilizing siRNA is to avoid off-target effects, which involve uncontrolled modulation of genes or pathways, leading to unnecessary silencing and alterations in cellular processes [41]. These off-target effects can range from minor changes in gene expression to more significant phenotypic consequences [42]. One of the critical factors influencing the extent of off-target effects is siRNA design, which requires rigorous computational tools and experimental validation strategies. Notably, in this study, by employing the novel strategy reverse to the current siRNA screening, tRF-T36 identified from our tRF library was addressed with no significant cytotoxicity on normal colon epithelial cells and exhibited no significant side effects *in vivo*, indicating that the putative binding sites on NUCKS1 mRNA utilized in the siRNA design might hold great promise for achieving low off-target effects. Moreover, further studies that focus on optimizing sequences based on the binding sites in NUCKS1 mRNA complementary to tRF-T36 could greatly enhance the efficacy of siRNAs designed to suppress colorectal cancer (CRC).

In conclusion, our study reports the first investigation on therapeutics targeting the potent oncogene NUCKS1 to suppress cancer progression. Based on our library of tRFs originated from medicinal plants, NUCKS1-orientated bioinformatics screening identified tRF-T36, a 5′ tRNA fragment of tRNA^ASN(GUU)^ of Chinese yew (*Taxus chinensis*), exhibiting stronger inhibitory effects than taxol against CRC progression. Mechanistically, tRF-T36 binds directly to the 3′ UTR of NUCKS1 mRNA to downregulate its expressions *via* RNAi pathway, which further inhibits the PI3K-Akt pathway. Collectively, our findings emphasize the importance of NUCKS1 as a promising druggable target for CRC. Furthermore, the present study provides the first siRNA sequence, tRF-T36 mimic, as small RNA drug candidate, thereby shedding light on CRC therapeutics.

## CRediT authorship contribution statement

**Kai-Yue Cao:** Writing – original draft, Validation, Methodology, Investigation. **Da Zhang:** Validation, Investigation. **Long-Bo Bai:** Validation, Investigation. **Tong-Meng Yan:** Validation, Investigation. **Yan Chen:** Validation. **Yu-Yang Jiang:** Writing – review & editing. **Zhi-Hong Jiang:** Writing – review & editing, Supervision, Funding acquisition, Conceptualization.

## 5 Limitations of the study

Although we have demonstrated that tRF-T36 mimic identified from Chinese yew inhibits CRC by targeting NUCKS1 to further downregulate PI3K-Akt pathway. However, the anti-metastatic effect of tRF-T36 mimic *in vivo* has not been carried out. Further research is needed on the downstream signaling of NUCKS1, as well as on the suppressive effect of this RNA species on other tumor cells. These will help to address more evidences in plant-derived RNAs treating diseases such as cancer.

## Declaration of competing interest

The authors declare that they have no known competing financial interests or personal relationships that could have appeared to influence the work reported in this paper.
