# Supplementary material for: Targeting NUCKS1 with a fragment of tRNAAsn(GUU) of Chinese yew for the treatment of colorectal cancer
Source: Noncoding RNA Res. 2024 Nov 12;11:38–47. doi: 10.1016/j.ncrna.2024.11.002 (PMC11683283; doi:10.1016/j.ncrna.2024.11.002)
Supplement: Multimedia component 1 [file mmc1.docx]

**Supplementary materials for**

**Targeting NUCKS1 with a fragment of tRNA^Asn(GUU)^ of Chinese yew for the treatment of colorectal cancer**

Kai-Yue Cao^1^, Da Zhang^1^, Long-Bo Bai^1^, Tong-Meng Yan^1^, Yan Chen^2^, Yu-Yang Jiang^2,3*^, Zhi-Hong Jiang^1*^

This file includes:

1. Supplementary Table S1

2. Supplementary Figures S1-S3

**Table S1. Primers for quantitative real-time PCR.**

| **Gene name** | **Forward (5'-3')** | **Reverse (5'-3')** |
| --- | --- | --- |
| NUCKS1 | TGCCCAAACCCAGACTAAAG | GACCCTTCATCCCCAGATTT |
| Caspase-3 | CATGGAAGCGAATCAATGGACT | CTGTACCAGACCGAGATGTCA |
| Caspase-6 | ATGGCGAAGGCAATCACATTT | GTGCTGGTTTCCCCGACAT |
| Caspase-7 | CGGTCCTCGTTTGTACCGTC | CGCCCATACCTGTCACTTTATCA |
| Caspase-8 | ACACAGTCGAGTAGACTCTCAAA | AGGAAGTGATGCTCGTTCAGA |
| Caspase-9 | CTCAGACCAGAGATTCGCAAAC | GCATTTCCCCTCAAACTCTCAA |
| Caspase-10 | TAGGATTGGTCCCCAACAAGA | GAGAAACCCTTTGTCGGGTGG |
| GAPDH | TGAAGGTCGGAGTCAACGGATT | CGTTCTCAGCCTTGACGGT |


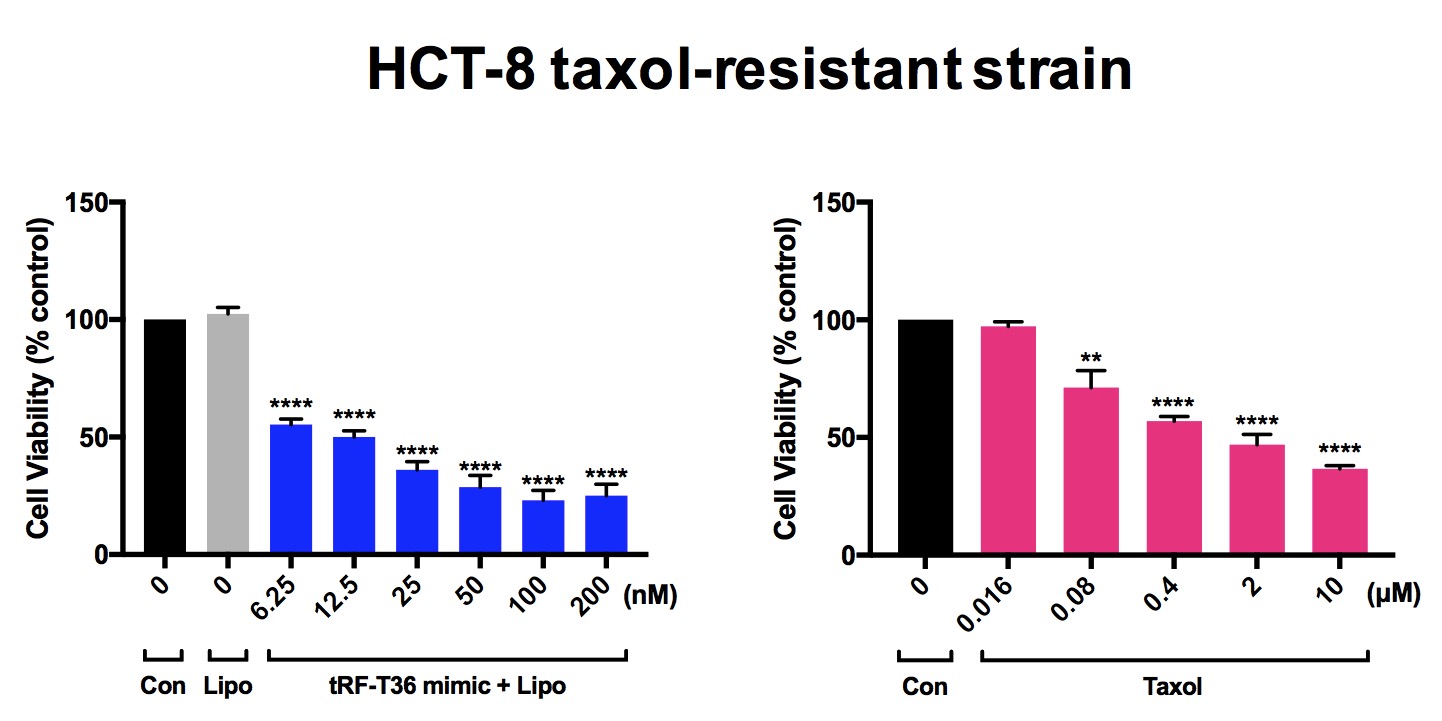


**Figure S1.** Dose-dependent investigations of tRF-T36 mimic (left) and taxol (right) on taxol-resistant HCT-8 strain**.**


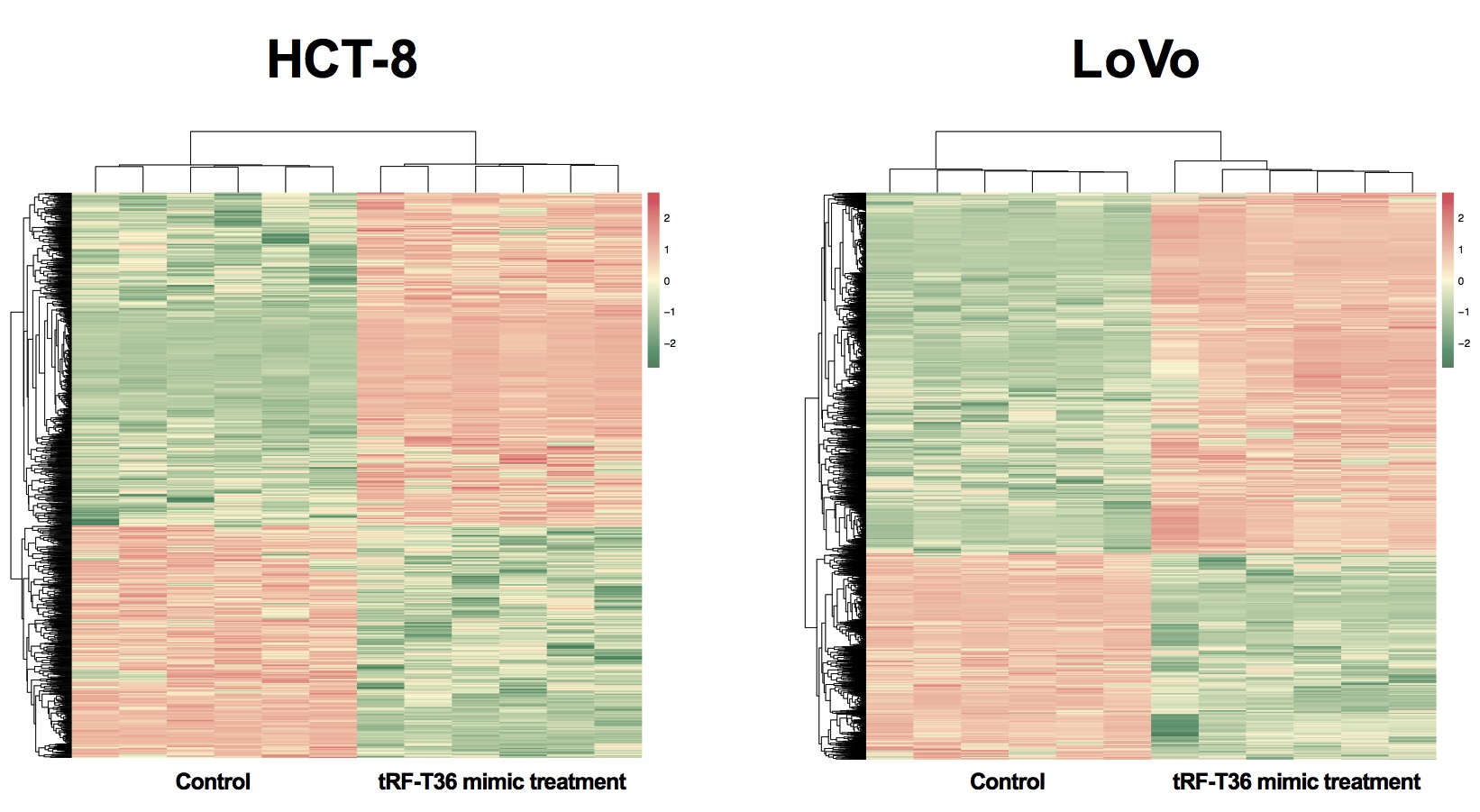


**Figure S2.** Hierarchical bi-clustering analysis of the significant gene signatures in CRC cells treated by tRF-T36 mimic and liposomes.


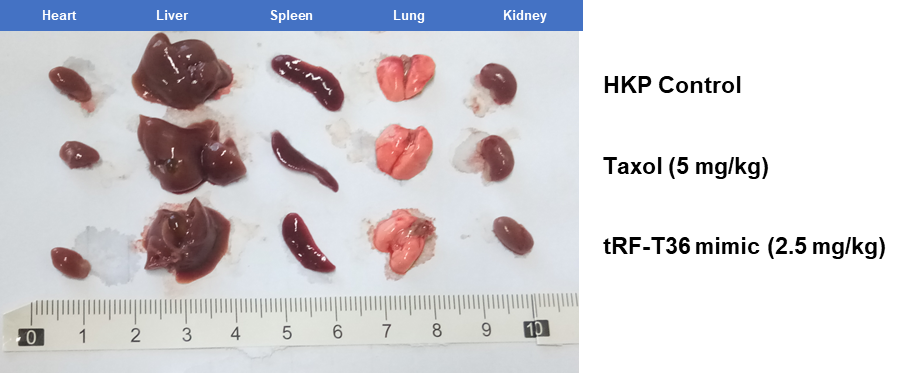


**Figure S3.** Major organs of HCT-8 xenograft nude mice treated by tRF-T36 mimic, taxol or HKP as control.
